# Impact of Ascorbate—Glutathione Cycle Components on the Effectiveness of Embryogenesis Induction in Isolated Microspore Cultures of Barley and Triticale

**DOI:** 10.3390/antiox10081254

**Published:** 2021-08-05

**Authors:** Iwona Żur, Przemysław Kopeć, Ewa Surówka, Ewa Dubas, Monika Krzewska, Anna Nowicka, Franciszek Janowiak, Katarzyna Juzoń, Agnieszka Janas, Balázs Barna, József Fodor

**Affiliations:** 1 The Franciszek Górski Institute of Plant Physiology Polish Academy of Sciences, Niezapominajek 21, 30-239 Kraków, Poland; p.kopec@ifr-pan.edu.pl (P.K.); e.surowka@ifr-pan.edu.pl (E.S.); e.dubas@ifr-pan.edu.pl (E.D.); m.krzewska@ifr-pan.edu.pl (M.K.); a.nowicka@ifr-pan.edu.pl (A.N.); f.janowiak@ifr-pan.edu.pl (F.J.); k.juzon@ifr-pan.edu.pl (K.J.); a.janas@ifr-pan.edu.pl (A.J.); 2 Plant Protection Institute, Centre for Agricultural Research, Herman Ottó út 15, 1022 Budapest, Hungary; barna.balazs@agrar.mta.hu (B.B.); fodor.jozsef@atk.hu (J.F.)

**Keywords:** antioxidant defence, ascorbate, glutathione, microspore embryogenesis, reactive oxygen species, *Hordeum vulgare*, ×*Triticosecale* Wittm.

## Abstract

Enhanced antioxidant defence plays an essential role in plant survival under stress conditions. However, excessive antioxidant activity sometimes suppresses the signal necessary for the initiation of the desired biological reactions. One such example is microspore embryogenesis (ME)—a process of embryo-like structure formation triggered by stress in immature male gametophytes. The study focused on the role of reactive oxygen species and antioxidant defence in triticale (×*Triticosecale* Wittm.) and barley (*Hordeum vulgare* L.) microspore reprogramming. ME was induced through various stress treatments of tillers and its effectiveness was analysed in terms of ascorbate and glutathione contents, total activity of low molecular weight antioxidants and activities of glutathione–ascorbate cycle enzymes. The most effective treatment for both species was a combination of low temperature and exogenous application of 0.3 M mannitol, with or without 0.3 mM reduced glutathione. The applied treatments induced genotype-specific defence responses. In triticale, both ascorbate and glutathione were associated with ME induction, though the role of glutathione did not seem to be related to its function as a reducing agent. In barley, effective ME was accompanied by an accumulation of ascorbate and high activity of enzymes regulating its redox status, without direct relation to glutathione content.

## 1. Introduction

Each stress that disturbs cellular homeostasis and oxygen metabolism leads to the generation of reactive oxygen species (ROS) and induces the so-called ‘oxidative stress’ [1]. Effective antioxidant protection in cells is needed for survival under stress conditions, especially for plants that do not have the possibility of the ‘fight-or-flight’ response.

However, ROS are no longer considered only the inevitable, toxic by-products of aerobic metabolism—their role proved to be essential in many life processes [1,2,3]. Depending on the subtle and dynamic homeostasis between production and scavenging, ROS can be considered as life-threatening or life-saving molecules, and the latter possibility is based on their involvement in stress signalling and initiation of defence reactions.

Our earlier studies [4,5] clearly showed that the initiation of microspore embryogenesis (ME) was also associated with ROS generation. This is fully justified as the process—highly resembling zygotic embryo formation in planta, although induced in immature male gametophyte cells in response to stress—involves very stressful procedures of anther or microspore isolation and transfer to in vitro culture conditions. Our recent results have suggested that ROS generation is not merely a side effect of stress treatment and a drastic change of the cell environment, but the first requirement for microspore reprogramming towards sporophytic development. At the same time, high activity of the antioxidant system is necessary for microspore survival and successful induction of embryogenic development. Furthermore, it was presumed that the next stages of embryo-like structure (ELS) development were also regulated by cellular redox homeostasis. The observed variability between two cereal species (barley and triticale) suggests that in addition to basic antioxidant enzymes, such as superoxide dismutase (SOD) and catalase (CAT), other enzymatic and non-enzymatic components of antioxidative defence are of great importance, including those involved in the ascorbate–glutathione cycle [5].

Ascorbate and glutathione are among the most important low molecular weight (LMW) antioxidants, and they are present in the majority of plant cells at high concentrations. Both molecules in the cellular environment are mostly found in reduced form, which enables them to interact with many cellular components and play a multifunctional role in plant metabolism, growth and development [6,7]. Both molecules scavenge ROS and are elements of the very important antioxidant Foyer–Halliwell–Asada pathway also known as the ascorbate–glutathione cycle. Reduced ascorbate (ASC) acts as an electron donor for ROS decomposition by ascorbate peroxidase (APX) in this cycle, and it is simultaneously oxidized to mono- and dehydroascorbate (MDHA, DHA). They can be reduced back in reactions catalysed by specific enzymes: monodehydroascorbate reductase (MDHAR) and dehydroascorbate reductase (DHAR) using reduced glutathione (GSH) and reduced nicotinamide adenine dinucleotide (NADH) as the source of electrons. The produced oxidized glutathione (glutathione disulphide, GSSG) is in turn reduced back with electrons transferred from nicotinamide adenine dinucleotide phosphate (NADPH) by glutathione reductase (GR). However, both antioxidants can also act independently and their functions in plant cells are generally determined by their cellular compartmentalization and intracellular environment. The role of glutathione in plant development, with particular emphasis on its function in plant embryo formation both in in vivo and in vitro systems, was discussed in details in our earlier reports [4,5]. Moreover, it plays a role in assimilation, transport and storage of sulphur as a thiol compound. It is also used to detoxify certain toxic electrophilic compounds (e.g., heavy metals, oxo-aldehydes or formaldehyde) by forming a reversible covalent bond in reactions catalysed by glutathione transferases, glyoxalases and formaldehyde dehydrogenase, or as a precursor of phytochelatins. The ratio of GSH to GSSG determines cell redox potential, and thus can regulate gene expression patterns, signalling as well as the synthesis and activity of some proteins [6,8].

In contrast, some data indicate that enhanced ROS generation has little effect on the reduced/oxidized ascorbate ratio. This is probably connected with the apoplastic location of DHA under oxidative stress (reviewed in [6]). It is possible that the intracellular ascorbate pool is largely maintained in a reduced state with a simultaneous increased DHA accumulation in the apoplast. This apoplastic fraction of ascorbate is mainly responsible for stress perception, signalling and defence initiation, while cytoplasmic ascorbate acts as a cofactor of several enzymes catalysing biosynthesis of phytohormones (abscisic acid, gibberellins, ethylene), pigments (anthocyanins) and secondary metabolites (flavonoids, glucosinolate) (reviewed by [6,9]). Ascorbate also regulates plant growth by controlling the biosynthesis of hydroxyproline-rich proteins involved in cell cycle progression, and by modulating the energetic state of plasmalemma and cell wall structure [10]. It is also involved in apical meristem formation, development of root architecture, regulation of flowering time and leaf senescence. Recent reports also postulated its participation in epigenetic regulation (review in [11]).

Several studies have indicated the possibility that ascorbic acid or glutathione application increases ME effectiveness. In the majority of cases, both compounds were supplemented to the induction or regeneration media, which resulted in enhanced ELS formation and green plant regeneration [12,13,14,15].

As mentioned above, recent results of our group [4,5] have suggested the involvement of various enzymatic or non-enzymatic components of antioxidant defence in ME regulation. Therefore, in the present study, we examined the role of the Foyer–Halliwell–Asada cycle in ME induction. Using two DH lines of triticale and two barley cultivars that significantly differed in ME responsiveness, we analysed the effect of various tiller stress pre-treatments on the effectiveness of ME induction, ROS generation, reduced and oxidized ascorbate and glutathione levels, as well as the total activity of LMW antioxidants and the activity of ascorbate–glutathione cycle enzymes: APX, DHAR, MDHAR and GR. The results broaden our understanding of the mechanisms determining ME effectiveness and the role of antioxidant system in the initiation of microspore reprogramming towards sporophytic development.

## 2. Materials and Methods

### 2.1. Plant Material and Growth Conditions

Two DH lines of winter triticale (×*Triticosecale* Wittm.): DH19 and DH28, derived from the F1 generation of a cross between the German inbred line ‘Saka 3006’ and Polish cv. ‘Modus’ and two cultivars of barley (*Hordeum vulgare* L.): winter cv. ‘Igri’ and spring cv. ‘Golden Promise’, highly differentiated in terms of ME responsiveness [16,17,18,19,20], were used in the study. The seeds of DH lines of triticale were obtained from the State Breeding Institute at the University of Hohenheim (Germany), whereas the seeds of barley cultivars were obtained from Leibniz Institute of Plant Genetics and Crop Plant Research (Gatersleben, Germany).

Winter forms of barley and triticale were vernalized for 7 weeks at 4 °C and 8/16 h day/night photoperiod in perlite moistened with Hoagland’s nutrient solution (HS; according to Wędzony [21]). Spring barley, cv. Golden Promise, was grown in the same conditions for 2 weeks. Subsequently, vernalized plantlets were transferred to a mixture of soil and sand (3/1; *v/v*) and grown at 20 °C and 16 h/8 h photoperiod. Additional illumination with an irradiance of 400 μmol m^−2^ s^−1^ was provided by high pressure sodium (HPS) lamps SON-T + AGRO (Philips, Brussels, Belgium) during unfavourable weather conditions.

The experiment was repeated twice, and microspore isolation was started each time in January (barley) and March (triticale).

### 2.2. ME Inducing Treatment, Microspore Isolation and In Vitro Culture Conditions for Antioxidant System Analysis

The procedure of ME induction was developed at the Plant Cell Biology Dept. IPP PAS as a combination of the requirements for triticale and barley, and was described in details in Żur et al. [5]. Briefly, tillers were harvested at the mid- to late uninucleate stage of microspore development—optimal for ME induction, placed in HS solution and stored for 3 weeks at a low temperature (4 °C) in the dark.

Four days before microspore isolation, tillers were transferred to fresh HS solution (control, low temperature treatment, LT), HS supplemented with 0.3 mM GSH (LT+GSH), HS supplemented with 0.3 M mannitol (LT+MAN) or HS supplemented with both GSH and mannitol (LT+MAN+GSH).

Depending on the plant species, about 20 (triticale) and from 20 to 60 spikes (for cv. ‘Igri’ and cv. ‘Golden Promise’, respectively) were used for one isolation procedure.

The microspore isolation procedure was previously described in Żur et al. [4,5]. After isolation, microspores were collected and sampled for in vitro culture and biochemical analysis.

Microspore suspensions with a density of approximately 70,000 microspores per ml (triticale) and 100,000 microspores per ml (barley) were cultured in the induction medium 190-2 [22], modified according to Pauk et al. [23], and KBP [24] for triticale and barley, respectively. Triticale microspores were co-cultured with simultaneously dissected ovaries (10 ovaries per 1 mL of suspension). All cultures were incubated in the dark at 26 °C.

### 2.3. Parameters Describing Productivity, Viability and Embryogenic Potential of Microspores

Microspore suspensions isolated from pre-treated tillers were evaluated using the following parameters:

(i) Microspore yield—mean number of isolated microspores obtained per one spike of a donor plant calculated using a Neubauer chamber.
(ii) Microspore viability—percentage of fully viable microspores in the whole population of isolated cells, assessed based on its reaction with fluorescein diacetate (FDA; 0.01%; λ_Ex_ = 465 nm, λ_Em_ = 515 nm, green fluorescence; according to Heslop-Harrison and Heslop-Harrison [25].

Microspore samples were collected at the end of the isolation procedure and examined using a Nikon Eclipse E600 epifluorescence microscope equipped with a Zyla 4.2 (Andor) camera and NIS-Elements AR 4.00 software. At least 500 microspores from 10 fields of view (magnification ×100) for each individual sample were analysed.

(iii) Effectiveness of ME induction—the number of developed embryo-like structures (ELS) observed after 6 weeks of in vitro culture, calculated per one spike of a donor plant [ELS/spike].

### 2.4. In Situ Histochemical Detection of Superoxide Anions and Hydrogen Peroxide in Microspores

Samples of isolated microspores were used for the detection of superoxide anions and hydrogen peroxide according to the protocols of Żur et al. [26] and Wohlgemuth et al. [27] with modifications.

Samples were collected with special handmade sieves prepared from pipette tips (5 mm in diameter) wrapped with 30 μm Nylon mesh (CellTrics, Sysmex Partec, Goerlitz, Germany). Sieves were placed in Eppendorf tubes in freshly prepared staining solutions containing 0.1% (*w/v*) nitroblue tetrazolium (NBT; for superoxide anion analysis) or 0.1% (*w/v*) 3,3′-diaminobenzidine-4-HCl (DAB; for hydrogen peroxide analysis) in potassium phosphate buffer (PPB, pH 7.0) containing 0.005% (*w/v*) Triton X-100. Then, the microspores were vacuum-infiltrated at room temperature (RT) for 120 min in the dark (NBT) or in the light (DAB). Afterwards, the microspores were treated with 50% (*v/v*) ethanol. Hydrogen peroxide was visualized as a reddish-brown colour, whereas superoxide anion through the conversion of NBT to blue formazan. Negative controls were in order to exclude false positive results.

Microscopic observations were performed under a Nikon Eclipse E600. Images were recorded using a digital camera (Nikon DS-Ri1, Tokyo, Japan) and processed with NIS-Elements AR 2.10 (Tokyo, Japan).

### 2.5. Low Molecular Weight Antioxidant Activity by DPPH Assay

To determine LMW antioxidant activity, microspores were incubated for 1 h at 26 °C in the dark, then frozen in liquid N_2_ and stored at −60 °C.

Plant material (microspores with a total FW of about 10 mg) was freeze-dried, ground with an MM400 ball mill (Retsch, Haan, Germany), and shaken for 2 h at room temperature (RT) after adding 1 mL of 50% ethanol. The extracts were then centrifuged for 20 min in a refrigerated centrifuge at 18,000× *g* (MPW-350R, Warszawa, Poland) and the supernatant was used for the measurements. Total antioxidant content (free radical scavenging activity) was measured using 0.5 mM solution of stable free radical 1,1-diphenyl-2-picrylhydrazyl (DPPH, SIGMA, Munich, Germany) in methanol according to the method by Brand-Williams et al. [28] with some modifications adapting the protocol to 96-well microtitre plates, and absorbance measurements in said microtitre plates [29]. Each sample was measured three times on separate plates by pipetting 50 μL of its supernatant with the addition of 250 μL of 0.5 mM DPPH solution into 3 wells for each measurement. After 30 min of reaction incubation at 37 °C, absorbance at 515 nm was determined using a Model 680 reader (Bio-Rad Laboratories, Hercules, CA, USA). Trolox was used as a standard in the following eight concentrations: 0.75, 1, 1.25, 1.5, 1.75, 2, 2.5, and 3 nM. 17.5 μL of each concentration was pipetted into three wells on each microtitre plate with the addition of 250 μL of 0.5 mM DPPH solution. Trolox equivalents for each measurement were calculated using the linear regression equation from a calibration curve representing linear relationships between absorbance and different Trolox concentrations. The results were expressed as μmoles of Trolox equivalents g^−1^ DW. For each DH line/cultivar and treatment, at least four measurements were performed on two independent samples collected from at least 6 different spikes.

### 2.6. Sample Preparation for Other Assays

The collected samples (1 mL of microspore suspension) were incubated for one hour at 26 °C in the dark, then centrifuged (2 min; 10,000× *g*; 4 °C) and washed with 50 mM Tris HCl buffer (pH 7.8) containing 1 mM EDTA-Na_2_. After the second centrifugation, pelleted microspores were used for further analysis. The activity of all antioxidant enzymes and the levels of reduced and oxidized forms of ascorbate and glutathione were detected spectrophotometrically in isolated microspore extracts using an Ultrospec 2100 pro UV/visible spectrophotometer (Amersham, Umeå, Sweden). At least three independent measurements were carried out for all assays.

### 2.7. Sample Preparation for Ascorbate and Glutathione Assays

Microspores were suspended in a five-fold (*v/w*) volume of ice-cold 6% meta-phosphoric acid and ground in liquid N_2_ in a mortar until the samples were thawed. After centrifugation (15 min, 12,000× *g*, 4 °C), the supernatant was used for the analyses.

### 2.8. Reduced and Oxidized Glutathione Measurements

Reduced (GSH) and oxidized (GSSG) glutathione were determined by an enzymatic recycling assay using GR according to Law et al. [30]. The procedure was described in detail in Żur et al. [4]. Briefly, the samples (50 μL of metaphosphoric acid extracts) were neutralised with 9 μL of 1.5 M triethanolamine. Total glutathione content was measured in an assay mix containing 59 μL of neutralized sample and 50 mM potassium phosphate buffer (pH 7.4), 2.5 mM EDTA-Na_2_, 1 mM 5,5′-dithio-bis(2-nitrobenzoic acid) (DNTB), 0.6 U of GR from baker’s yeast, 0.2 mM NADPH in a total volume of 600 μL. Total glutathione content was estimated by recording the absorbance at 412 nm. GSSG measurement was carried out as for total glutathione, except that GSH was derivatised by adding 2 μL of 2-vinylpyridine to the neutralized samples and incubated for 1h at RT. Total glutathione and GSSG contents were estimated from the standard curves based on a dilution series of GSH and GSSG in 6% metaphosphoric acid. GSH was calculated as the difference between total glutathione and GSSG concentrations.

### 2.9. Reduced and Oxidized Ascorbate Measurements

Reduced ascorbate (ASC) concentration was determined using the method of Foyer et al. [31]. The samples (62.5 μL metaphosphoric acid extracts) were neutralized with 7.5 μL of 1.5 M triethanolamine and 75 μL of 150 mM sodium phosphate buffer (pH 7.4) and subsequently mixed with 500 μL of 100 mM sodium phosphate buffer (pH 5.6). To oxidize ASC, 1 U ascorbate oxidase (AO) from *Cucurbita* sp. (Sigma-Aldrich, Saint Louis, MO, USA) was added to the assay mix. ASC content was determined based on the difference between the initial and final absorbance measured at 265 nm. Total ascorbate levels were determined after dehydroascorbate (DHA) reduction to ascorbate using dithiothreitol (DTT). A 10 mM DTT solution (37.5 μL) was added to the reaction mixture, prior to the addition of sodium phosphate buffer (pH 5.6) and AO, and incubated for 15 min at RT. Ascorbate concentration was calculated using the extinction coefficient of ascorbic acid (14.7 mM^−1^ cm^−1^). DHA content was obtained from the difference between total and reduced ascorbate concentrations.

### 2.10. Sample Preparation and Enzyme Activity Assays

Microspores were suspended in ice-cold 50 mM Tris HCl buffer (pH 7.8) containing 1 mM EDTA-Na_2_, 3% (*w/v*) soluble polyvinylpyrrolidone K25 and 0.5 mM Pefabloc SC protease inhibitor (Roche, 11429876001), and ground in liquid N_2_ in a mortar until thawed. After centrifugation (15 min, 12,000× *g*, 4 °C), the supernatant was used for the assays.

APX activity was detected according to the method of Nakano and Asada [32]. The assay mixture (570 μL) contained: 50 mM Tris-HCl buffer (pH 7.8), 0.25 mM ascorbic acid, 0.5 mM H_2_O_2_ and 20 μL of enzyme extract. Ascorbic acid oxidation was followed by measuring the absorbance at 290 nm, and APX activity was calculated using the extinction coefficient of ascorbic acid (2.8 mM^−1^ cm^−1^). A control reaction was carried out for each sample in the absence of H_2_O_2_. The enzyme activity was expressed in nmol ASC mg^−1^ protein min^−1^.

DHAR activity determination was based on the method of Klapheck et al. [33] by measuring the absorbance at 265 nm following DHA reduction. The assay mixture consisted of 30 μL of enzyme extract, 550 μL of 50 mM sodium phosphate buffer (pH 6.5), 1 mM EDTA-Na_2_, 1 mM GSH and 0.5 mM DHA. Reaction mixture without extract was used as a negative control. DHAR activity was calculated using the extinction coefficient of ascorbic acid (14.7 mM^−1^ cm^−1^) and expressed in nmol ASC mg^–1^ protein min^–1^.

MDHAR activity was estimated by measuring the decrease in absorbance at 340 nm due to NADH oxidation [34]. The reaction mixture consisted of 30 μL of enzyme extract, 575 μL of 50 mM Tris-HCl buffer (pH 7.8), 1 mM ASC, 0.1 mM NADH and 0.2 U AO. A control reaction was carried out for each sample in the absence of AO. MDHAR activity was calculated using the extinction coefficient of NADH (6.2 mM^−1^ cm^−1^) and expressed in nmol NADH mg^−1^ protein min^−1^.

GR activity was assayed by monitoring NADPH oxidation at 340 nm according to the method of Klapheck et al. [33]. The assay mixture and) consisted of 600 μL 50 mM Tris-HCl buffer (pH 7.8), 0.1 mM NADPH, 5.8 mM GSSG and 30 μL of enzyme extract. Control reactions were conducted in the absence of GSSG. GR activity was calculated using the extinction coefficient of NADPH (6.2 mM^−1^ cm^−1^) and expressed in nmol NADPH mg^−1^ protein min^−1^.

Enzyme activities were normalized to soluble protein content of microspore homogenates. Protein content in enzyme extracts was determined according to Bradford [35] using bovine serum albumin as a standard.

### 2.11. Statistical Analysis

All data after testing for normal distribution using the Shapiro–Wilk test were subject to two-way analysis of variance (ANOVA) followed by post hoc comparison using Duncan’s multiple range test (*p* ≤ 0.05). Variables with non-normal data distribution were analysed using non-parametric Kolmogorov–Smirnov tests (*p* ≤ 0.001). All statistical analyses were performed using the STATISTICA package version 12 (Stat Soft Inc., Tulsa, OK USA).

## 3. Results

### 3.1. The Effect of Tiller Pre-Treatments on the Effectiveness of ME Induction

Our results confirmed a significant variation in ME effectiveness between the two studied cereal species, as well as the intraspecific differentiation between two DH triticale lines and two barley cultivars (Table 1).

The results shown in Table 1 clearly indicated that microspore yield obtained from a single spike after isolation was almost 4-fold higher in triticale compared to barley. In this respect, the number of microspores obtained from DH19 (about 105,000 per spike) was significantly higher than that of DH28 (about 89,000 per spike), whereas the process of microsporogenesis was similarly effective in the studied barley cultivars, producing an average of 26,500 microspores per spike. At the same time, variation in microspore yields after various tiller pre-treatments was insignificant.

In contrast, microspore viability was higher in microspore suspensions of both barley cultivars (51–65%) in comparison to triticale (6–30%; Table 1). Moreover, the number of fully viable cells was higher in both responsive genotypes (DH28 and cv. Igri) in comparison to recalcitrant ones (DH19 and cv. Golden Promise). Particularly low cell viability (approximately 12%) was found in isolated DH19 microspores. Tiller pre-treatment with MAN resulted in a lower number of viable cells in triticale, although a decrease in the number of fully viable cells from about 30% to less than 18% in DH28 was a statistically significant effect.

Unfortunately, the number of ELS produced in isolated microspore cultures of triticale was relatively low (Table 1), not only in the standard recalcitrant DH19, but also in the usually moderately responsive DH28. The best effect was obtained in the combination of the LT treatment with MAN and GSH application (LT+MAN+GSH). This was the only treatment that allowed any ELS formation in DH19, although the effectiveness was below 1 ELS per spike. The results obtained with highly recalcitrant spring barley (Golden Promise) were only slightly better after the LT treatment in combination with MAN or after MAN and GSH application. The positive effects of LT+MAN and LT+MANGSH treatments could also be observed in isolated microspore cultures of cv. Igri characterized by a high embryogenic potential (Table 1).

### 3.2. In Situ Histochemical Detection of Superoxide Anions and Hydrogen Peroxide in Microspores

Superoxide anion and hydrogen peroxide generation was observed in isolated microspores of triticale and barley after all types of tiller pre-treatments. It can be seen that both superoxide anion (Figure S1) and hydrogen peroxide (Figure S1D–F) were accumulated mainly in the cell cytoplasm. In general, the signal was diffused but, in some areas, particularly near the nucleus enhanced ROS generation was detected. It was observed more often it the case of superoxide anion.

### 3.3. Total Activity of Low Molecular Weight Antioxidants in Microspores after Various ME-Inducing Pre-Treatments of Tillers

Total activity of LMW antioxidants was estimated using a method based on the scavenging capacity of a stable free radical (DPPH) compared to antioxidant capacity of Trolox, used as a reference. We found significant variation between various plant genotypes, tiller pre-treatments and their interactions (Table 2). Antioxidant activity also depended on tiller pre-treatments and was significantly higher in microspores isolated from tillers treated with LT+GSH (3.2 μM Trolox g^−1^ DW) and LT+MAN+GSH (2.9 μM Trolox g^−1^ DW) in comparison to other treatments (2.1−2.3 μM Trolox g^−1^ DW). The LT+GSH treatment was particularly effective for the responsive DH28 and cv. Igri.

**Figure 1:**
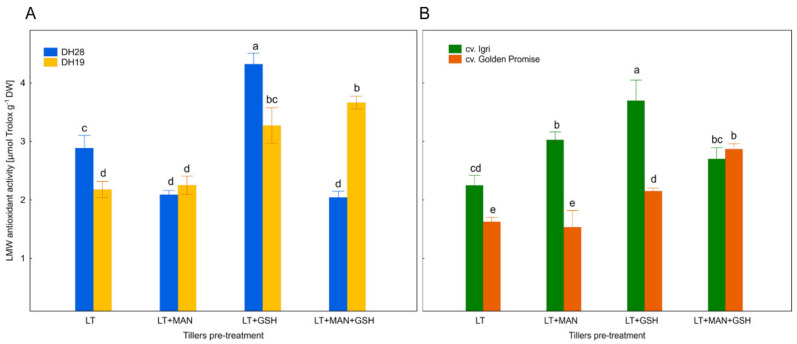
Total activity of low molecular weight (LMW) antioxidants in microspores of (**A**) two DH triticale lines (responsive DH28 and recalcitrant DH19) and (**B**) two barley cultivars of (responsive cv. Igri and recalcitrant cv. Golden Promise) isolated after various tiller pre-treatments. Data represent means of at least four measurements of two independent samples, each collected from at least 6 different spikes ± SE. Values marked with the same letter do not differ significantly according to Duncan’s multiple range test (*p* ≤ 0.05). Please see Figure 3, LT—low temperature tiller pre-treatment (21 days at 4 °C); LT+MAN—low temperature tiller pre-treatment combined with the application of 0.3 M mannitol; LT+GSH—low temperature tiller pre-treatment combined with the application of 0.3 mM reduced glutathione; LT+MAN+GSH—low temperature tillers pre-treatment combined with the application of 0.3 M mannitol and 0.3 mM reduced glutathione.

It was observed that antioxidant activity was in most cases significantly higher in microspores of responsive genotypes (DH28, cv. Igri) than in the recalcitrant DH19 and cv. Golden Promise (Figure 1A,B). The only exception was the LT+MAN+GSH treatment associated with enhanced activity of LMW antioxidants in microspores of both DH19 and cv. Golden Promise. The same treatment had no effect or reduced total antioxidant activity in microspores of cv. Igri and DH28. Only responsive genotypes were affected by the LT+MAN treatment, but its effect was genotype-specific and both increased and decreased antioxidant activity was detected in microspores of cv. Igri and DH28, respectively.

### 3.4. Glutathione and Ascorbate Levels and Their Reduction State in Triticale and Barley Microspores after Various ME-Inducing Pre-Treatments of Tillers

**Figure 2:**
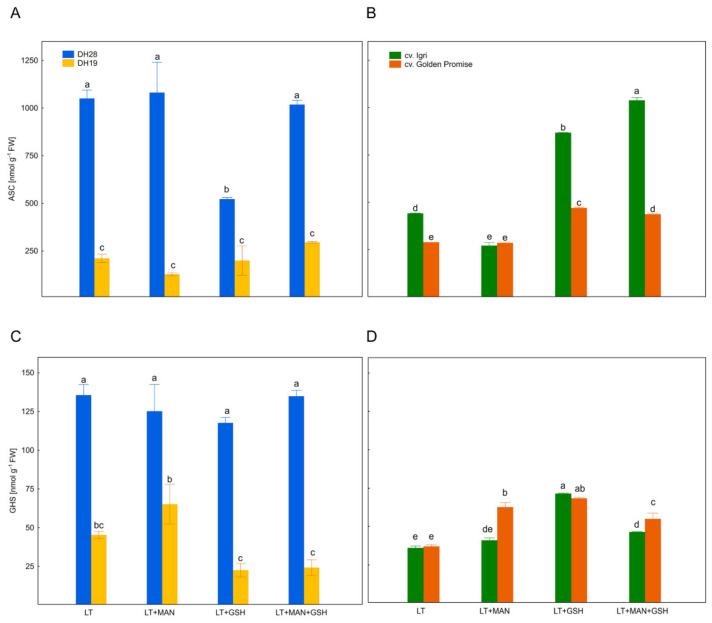
Reduced ascorbate (ASC) (**A**,**B**) and reduced glutathione (GSH) (**C**,**D**) contents in microspores of two DH triticale lines (responsive DH28 and recalcitrant DH19) and two barley cultivars (responsive cv. Igri and recalcitrant cv. Golden Promise) isolated after various tiller pre-treatments. Data represent means of three biological replications ± SE. Values marked with the same letter do not differ significantly according to Duncan’s multiple range test (*p* ≤ 0.05). Please see Figure 3, LT—low temperature tiller pre-treatment (21 days at 4 °C); LT+MAN—low temperature tiller pre-treatment combined with the application of 0.3 M mannitol; LT+GSH—low temperature tiller pre-treatment combined with the application of 0.3 mM reduced glutathione; LT+MAN+GSH—low temperature tillers pre-treatment combined with the application of 0.3 M mannitol and 0.3 mM reduced glutathione.

The level of ASC in microspores varied from 208 to 918 nmol g^−1^ FW (Figure 2A,B) and was significantly affected by plant genotype, tiller pre-treatment and their interactions (Table 2). Compared to ASC, GSH was detected in a significantly lower amount, ranging from 39 to 128 nmol g^−1^ FW (Figure 2C,D) and its content was influenced by plant genotype, but not tiller pre-treatment (Table 2). The effect of tiller pre-treatments also depended on the plant species and individual DH line/cultivar.

**Figure 3:**
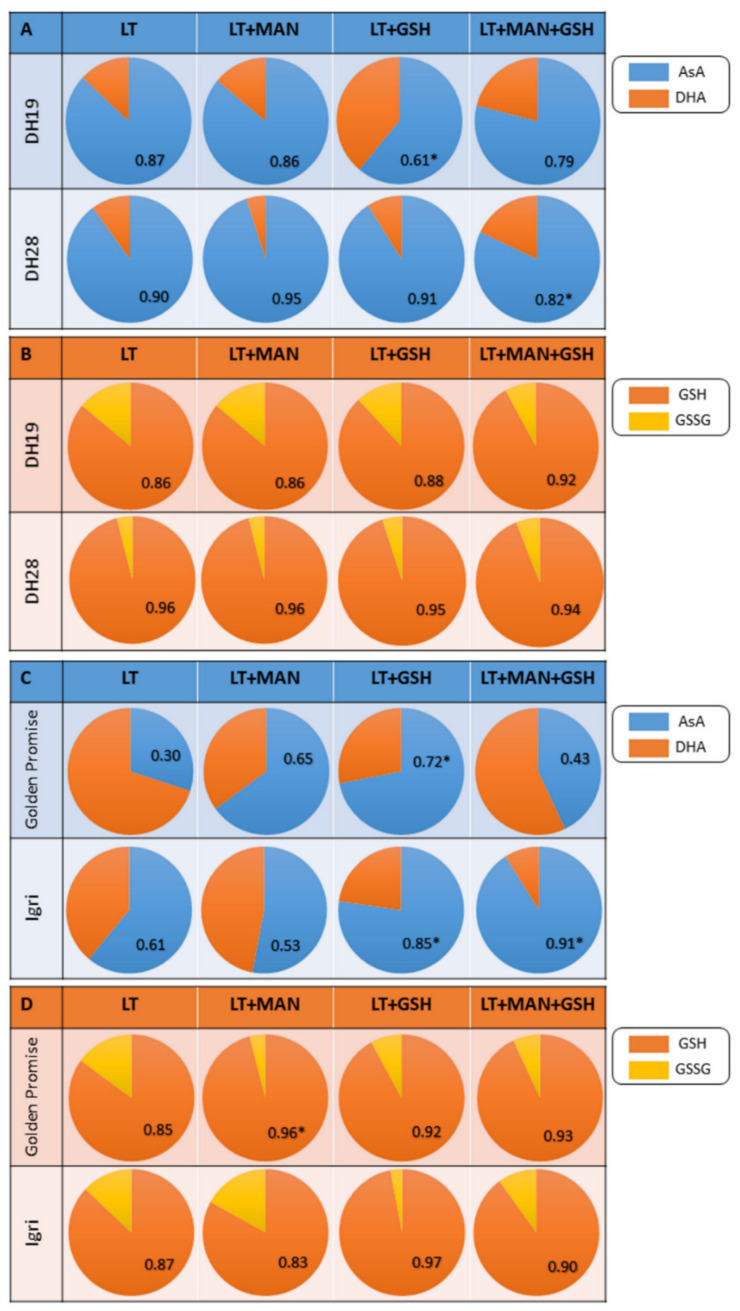
The proportion reduced (ASC, GSH) and oxidized (DHA, GSSG) forms of ascorbate and glutathione in their total pools in microspores of two DH triticale lines (responsive DH28 and recalcitrant DH19) (**A**,**B**) and two barley cultivars (responsive cv. Igri and recalcitrant cv. Golden Promise) (**C**,**D**) isolated after various tiller pre-treatments. The numbers represent the redox status of ascorbate (ASC/ASC+DHA) and glutathione (GSH/GSH+GSSG). Values significantly different from control (LT) according to the Kruskal–Wallis test (* *p* ≤ 0.001) are marked with an asterisk. LT—low temperature tiller pre-treatment (21 days at 4 °C); LT+MAN—low temperature tiller pre-treatment combined with the application of 0.3 M mannitol; LT+GSH—low temperature tiller pre-treatment combined with the application of 0.3 mM reduced glutathione; LT+MAN+GSH—low temperature tillers pre-treatment combined with the application of 0.3 M mannitol and 0.3 mM reduced glutathione.

In general, variation in ASC and GSH levels, as well as the redox potential of ascorbate (ASC/ASC+DHA) and glutathione (GSH/GSH+GSSG) was low in triticale microspores (Figure 2A,C and Figure 3A,B). Only the LT+GSH treatment resulted in a significant decrease in ASC level in microspores of DH28 in comparison to control (LT, Figure 2A). However, this effect did not change the redox status of ascorbate (Figure 3A). On the contrary, the LT+MAN+GSH treatment did not affect ASC content, but was associated with a lower ascorbate redox potential (Figure 2A and Figure 3A). This parameter was also relatively low in DH19 microspores after the LT+GSH treatment.

Two treatments (LT+GSH and LT+MAN+GSH) stimulated the accumulation of ASC in both barley cultivars and changed the ascorbate redox state (ASC/ASC+DHA) in microspores of one or both cultivars (Figure 2B; Figure 3C). The effect of the LT+MAN treatment was genotype-specific as it reduced ASC accumulation exclusively in microspores of cv. Igri. All treatments increased GSH content in microspores of cv. Golden Promise (Figure 2D), but only LT+MAN application also increased the glutathione redox potential (GSH/GSH+GSSG; Figure 3D). Similarly, an increased level of GSH was detected in microspores of cv. Igri after LT+GSH and LT+MAN+GSH treatments (Figure 2D), but it was not associated with a change in the GSH/GSH+GSSG ratio (Figure 3D).

### 3.5. The Activity of Enzymes of the Glutathione–Ascorbate Cycle in Microspores after Various ME-Inducing Pre-Treatments of Tillers

The activity of APX, which catalyses H_2_O_2_ reduction via ASC oxidation to monodehydroascorbate (MDHA) significantly depended on plant genotype, tiller pre-treatment and their interactions (Table 2). The same sources of variation were found to be statistically significant for all other enzymes (MDHAR, DHAR, and GR).

**Figure 4:**
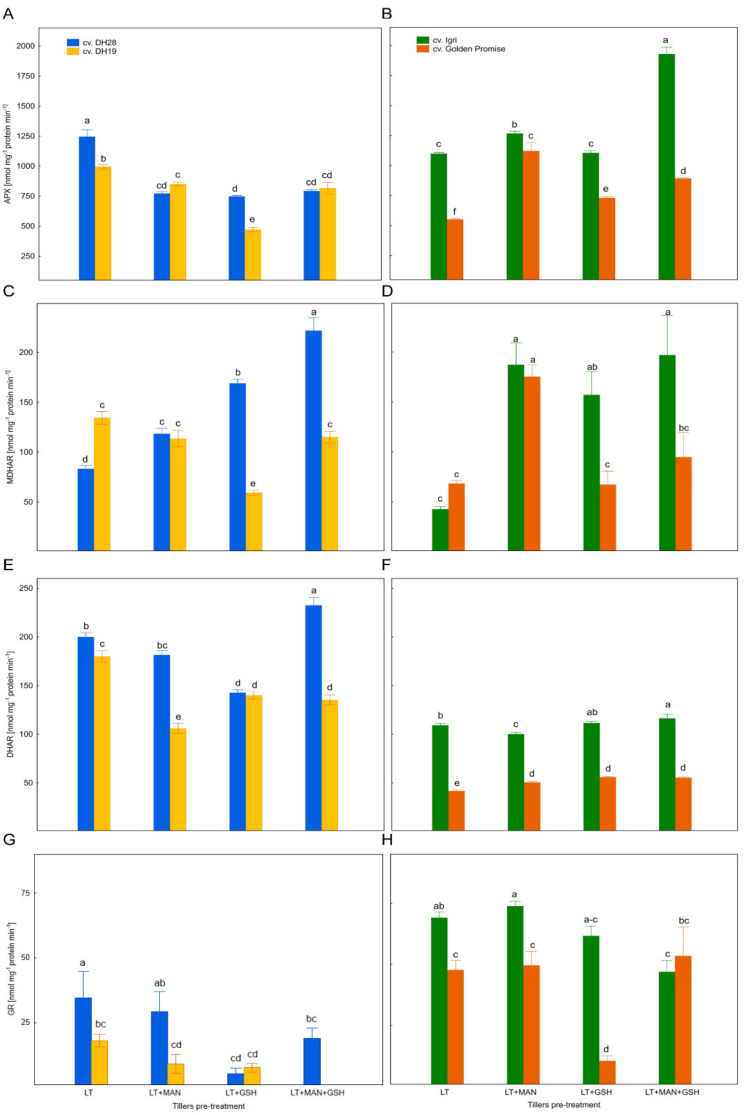
Activity of enzymes of the ascorbate–glutathione cycle: (**A**,**B**) ascorbate peroxidase (APX), (**C**,**D**) monodehydroascorbate reductase (MDHAR), (**E**,**F**) dehydroascorbate reductase (DHAR), (**G**,**H**) glutathione reductase (GR) in microspores of two DH triticale lines (responsive DH28 and recalcitrant DH19) and two barley cultivars (responsive cv. Igri and recalcitrant cv. Golden Promise) isolated after various tiller pre-treatments. Data represent means of three biological replications ± SE. Values marked with the same letter do not differ significantly according to Duncan’s multiple range test (*p* ≤ 0.05). Please see Figure 3, LT—low temperature tiller pre-treatment (21 days at 4 °C); LT + MAN—low temperature tiller pre-treatment combined with the application of 0.3 M mannitol; LT + GSH—low temperature tillers pre-treatment combined with the application of 0.3 mM reduced glutathione; LT + MAN + GSH—low temperature tillers pre-treatment combined with the application of 0.3 M mannitol and 0.3 mM reduced glutathione.

It should be noted that the average APX activity was significantly higher in the ME-responsive DH28 and cv. Igri in comparison to the recalcitrant DH19 and cv. Golden Promise (Figure 4A,B).

All modified tiller treatments resulted in a lower APX activity in triticale microspores of both DH lines (Figure 4A) compared to the LT treatment, with the strongest effect of LT+GSH application. In contrast, all modified treatments increased APX activity in microspores of cv. Golden Promise and two of them (LT+MAN and LT+MAN+GSH) increased APX activity in microspores of cv. Igri (Figure 4B). Similar activities of this enzyme were detected in microspores of both barley cultivars after the LT+MAN treatment and in both DH triticale lines after LT+MAN and LT+MAN+GSH treatments.

The activity of MDHAR, which can reduce MDHA to ASC, was significantly higher after all modified tiller treatments in microspores of two responsive genotypes (DH28 and cv. Igri), but the effect of the LT+MAN+GSH treatment was especially prominent in comparison to control (LT; Figure 4C,D). A higher activity of this enzyme was detected in microspores of cv. Golden Promise only after the LT+MAN treatment, whereas the only change observed in DH19 microspores was a decrease in MDHAR activity after the LT+GSH treatment.

The produced MDHA is further oxidized to DHA, which can be converted back to ASC in the reaction catalysed by DHAR. In this reaction, GSH is used as an electron donor that is simultaneously oxidised to GSSG. Generally, modifications of tiller pre-treatment applied in this study resulted in a decrease of DHAR activity in triticale microspores, with the exception of DH28 tillers, which showed significantly increased DHAR activity after the LT+MAN+GSH pre-treatment (Figure 4E). On the other hand, all treatment modifications resulted in a slight but significant increase in DHAR activity in microspores of cv. Golden Promise, whereas a slight increase (after LT+MAN+GSH) as well as a slight decrease (after LT+GSH) could also be observed in DHAR activity in cv. Igri microspores. The activity of this enzyme was almost always higher in ME-responsive genotypes in comparison to recalcitrant ones (Figure 4E,F).

In comparison with other enzymes of the glutathione–ascorbate cycle, the average activity of GR which catalyses the conversion of GSSG to its reduced form, was low in triticale microspores, especially in the ME-recalcitrant line DH19 (8.7 nmol mg^−1^ protein min^−1^). Microspores of DH28 showed more than twice higher average GR activity (21 nmol mg^−1^ protein min^−1^) as compared to DH19. Similarly, the average GR activity in microspores of ME-recalcitrant cv. Golden Promise was about 1.5-fold lower in comparison to responsive cv. Igri (40.3 versus 62.9 nmol mg^−1^ protein min^−1^). Among various tiller pre-treatments, LT+MAN+GSH was associated with a decreased GR activity in microspores of both triticale DH lines and barley cv. Igri (Figure 4G,H). The activity of GR in DH19 fell below the level of detection. Strikingly, the LT+GSH treatment resulted in a large decrease in GR activity in microspores of barley cv. Golden Promise and in both DH triticale lines. Variation in the reactions between responsive and recalcitrant genotypes was observed after the LT+MAN treatment in both species and after the LT+GSH treatment in barley (Figure 4G,H).

## 4. Discussion

The process of ME is both scientifically fascinating as a manifestation of cell totipotency and commercially important due to the possibility of instant production of completely homozygous doubled haploid (DH) plants. DH technology complements conventional plant breeding and can be used in areas such as genome engineering and gene mapping, often applied for substantial crop improvement [36,37,38,39]. However, the possibility of its application on a commercial scale is often limited by the low effectiveness of ME induction, which is strongly dependent on plant genotype, environmental conditions and complex system of interactions between internal and external factors.

Substantial progress in recent years has significantly increased our knowledge about ME mechanism and its regulation, but many issues remain unexplored or unclear. The use of stress treatments to induce efficient microspore reprogramming was a very important discovery [40]. In addition to being a trigger redirecting the pathway of development, stress also exerts a considerable adverse effect on cells, which can result in their death or genome rearrangements [41]. Thus, the type, intensity and duration of stress treatment must be carefully optimized to the requirements of each individual genotype and its stress tolerance. Prior experimental data suggested that exposure to certain mild primary stresses (e.g., LT, conventionally used as an ME-inducing factor in many plant species) can activate defence mechanisms and increase tolerance to more intensive stresses that occur during microspore isolation and transfer to in vitro culture conditions [42]. Such a cross-tolerance phenomenon is well known in plants, and in many cases, it is associated with increased ROS generation, which may also serve as signalling molecules activating cellular defence responses [43]. Several studies have confirmed that ME initiation is associated with the upregulation of genes encoding enzymes involved in antioxidative defence, as well as increased activity and/or accumulation of LMW antioxidants (reviewed in [20,44,45] and references therein). Therefore, different types of antioxidants were added to media used for in vitro cultures in order to achieve positive effects on ELS formation and plant regeneration ([5] and references therein).

The main aim of our research has been to determine the role of ROS and antioxidant defence in microspore reprogramming and ME induction. Our earlier investigations concerning several DH triticale lines significantly differed in ME effectiveness. Żur et al. [26] not only confirmed ROS generation in anthers after LT tiller pre-treatment, but also suggested that a certain threshold level of ROS is required for ME initiation; however, ROS generation had to go hand in hand with an efficient antioxidant defence for effective reprogramming of microspores. Furthermore, the effects of GSH application during the LT tiller pre-treatment [4] suggested its dual role: protection of microspores from oxidative stress and stimulation of ELS development, probably through modulation of cellular redox homeostasis. Our data indicated that intensive ROS elimination could suppress the signal necessary for microspore reprogramming [4]. To test this hypothesis in a broader range of taxa, the present study, in addition to two DH triticale lines (DH19 and DH28), also used two barley cultivars markedly heterogeneous in terms of ME capacity (Igri and Golden Promise) [16,17,18,19,20]. The experimental work was also extended to include tiller pre-treatments with MAN, a sugar alcohol that is often used to simulate osmotic stress and commonly applied for ME induction in barley. A strong positive correlation (*r* = 0.85) between the generation of hydrogen peroxide (H_2_O_2_) and ME effectiveness confirmed the important role of ROS in microspore reprogramming [5]. It was also revealed that the efficiency of ME induction in both triticale and barley was significantly modified by seasonal effects associated with fluctuations in the activity of the main antioxidant enzymes—SOD and CAT [5]. The observed genetic specificity of antioxidant defence triggered in response to various ME-inducing treatments suggested an important role of other enzymatic or non-enzymatic elements of antioxidative defence. Continuing our investigations, in the present study, we examined cellular ROS generation, the role of the main LMW antioxidants (ascorbate and glutathione) and enzymes involved in the ascorbate–glutathione cycle in determining the effectiveness of ME induction.

The effects of various ME-inducing stress pre-treatments of tillers were assessed by analysing several parameters related to isolated microspore condition, antioxidant activity and embryogenic potential. In line with previous results [5], the analysis of microspore yield confirmed that barley spikes produced a lower number of microspores in comparison to triticale and that the number of produced microspores was not significantly affected by stress pre-treatments. The low viability of microspores detected in the population of triticale microspores isolated in early spring (March) was also consistent with earlier observations [5]. It was probably the main cause of low effectiveness of ELS formation in isolated microspore cultures of DH28, which is usually significantly more productive [4,5,19,26]. Interestingly, the negative effect of MAN on triticale microspore viability was associated with a higher effectiveness of ELS production, which also confirmed previously published results [5].

Low effectiveness of ME induction in microspore suspension of cv. Golden Promise probably resulted from the specificity of tiller pre-treatments, not sufficiently meeting the requirements of this genotype. On the contrary, highly successful ME initiation in isolated microspore cultures of cv. Igri indicated a significant difference in response to various tiller pre-treatments (observed only as a tendency in other plant materials tested). The observed positive effect induced by the combined LT+MAN+GSH treatment on ELS formation was also in accordance with earlier results on triticale and rye [5,46].

In contrast to animal cells, in which ROS generation is associated mainly with mitochondrial activity, ROS biogenesis in plant cells can be induced by multiple metabolic processes in all cellular compartments [3]. Depending on the cell origin, phase of development and environmental conditions, ROS generation was detected in the plant cell wall, apoplast, plasma membrane, cytoplasm, and organelles involved in oxygen metabolism [47]. However, in light-deprived or non-green tissues (cells) such as microspores, the mitochondrion is postulated to be the major site of ROS production. Mitochondria-originating ROS affect many cell functions through various signalling cascades, retrograde signalling, and interaction with plant hormones [48]. However, the involvement of other ROS-generating pathways cannot be excluded. For example, ROS generation in the cell wall, apoplast, or plasma membrane plays an important role in the interactions between the plant and its environment as well as in plant development [3]. The diffuse patterns of superoxide anion and hydrogen peroxide distribution in the microspore’s cytoplasm observed in our study suggest, that microspore isolation and transfer to in vitro culture conditions induced oxidative stress of high intensity in various cell compartments or that the induced signal was transported across the cell area. The more intense accumulation of superoxide anion observed near the nucleus seems to confirm its role in transmitting the signal to the nucleus, which probably leads to an activation of various redox signalling pathways [49]. It could be supposed that the strong NBT staining is probably related to extensive oxidative damage near the nucleus, and thus the modification of some nuclear factors followed by changes in the expression of genes linked to antioxidant responses, cellular transformation, and cell proliferation [3,49]. All these events ending finally in ME induction in the presented model.

The results of recalcitrant genotypes (DH19, cv. Golden Promise) suggested that the high total activity of LMW antioxidants might be one of the factors important for ME induction. It could be attributed to the compensation of inefficient enzymatic defence [5,26]. Such high activity of LMW antioxidants was not necessary for microspores of responsive genotypes equipped with more active antioxidant enzymes [5]. Furthermore, the relatively high total activity of LMW antioxidants induced by GSH pre-treatment of tillers was associated with reduced effectiveness of ELS formation in responsive DH lines/cultivars.

**Figure 5:**
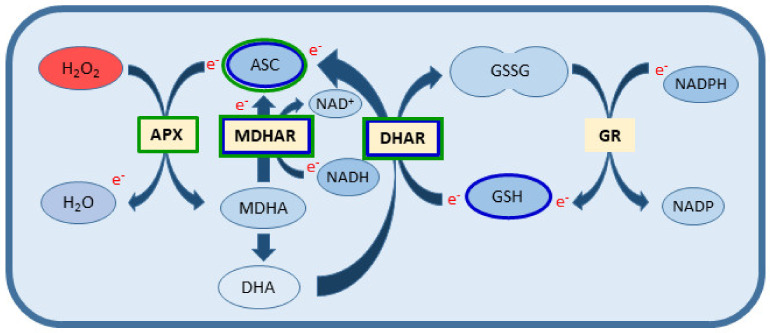
Summary of the work showing the role of the ascorbate–glutathione cycle in the scavenging of ROS (H_2_O_2_) generated during ME-induction in microspores of the responsive triticale line (DH28) and barley cultivar (cv. Igri). The generated H_2_O_2_ is initially reduced via the oxidation of reduced ascorbate (ASC) in a reaction catalysed by APX. Two MDHA molecules spontaneously disproportionate into ASC and dehydroascorbate (DHA). Both MDHA and DHA can be reduced back to ASC in reactions catalysed by MDHAR and DHAR, where GSH and NADH are used as electron donors. Oxidized glutathione (GSSG) is converted back to its reduced form by GR using NADPH as an electron donor. Ascorbate–glutathione cycle components, which are largely involved in the process of ME-induction in triticale and barley are marked with the following colour outlines: dark blue for triticale and green for barley. APX—ascorbate peroxidase; ASC—reduced ascorbate; DHA—dehydroascorbate; DHAR—dehydroascorbate reductase; GR—glutathione reductase; GSH—reduced glutathione; GSSG—oxidized glutathione, glutathione disulphide; MDHA—monodehydroascorbate; MDHAR—monodehydroascorbate reductase; NAD(P)H/NAD(P)+—nicotinamide adenine dinucleotide (phosphate) reduced/oxidized; e^−^—transferred electrons.

Based on the results, it can be concluded that the embryogenic potential of triticale seems to be associated with ascorbate and glutathione metabolism (Figure 5). Such a conclusion could be drawn by comparing the level of ASC and GSH accumulation in microspores of responsive and recalcitrant DH lines. The responsive DH28 line was also characterized by high activity of MDHAR and DHAR, enzymes that catalyse the conversion of oxidised ascorbate (MDHA and DHA) to a reduced form (ASC). However, the relatively low activity of APX in triticale microspores after the most effective ME-inducing treatments suggests that reduced ascorbate may play other roles than antioxidative in microspore reprogramming. The role of GR is questionable, because its activity, although relatively low in DH28 microspores, was significantly higher in comparison to recalcitrant DH19. Contrary to the previous report [4], exogenous GSH application during the LT pre-treatment of tillers did not improve ELS formation in isolated microspore cultures of the responsive DH28 line. Such a divergent effect of GSH was also observed earlier [5], and then it was explained as a result of excessive decomposition of H_2_O_2_, whose amount in the cells declined below the threshold necessary to initiate microspore reprogramming.

In microspores of highly responsive barley (cv. Igri), the most effective ME-induction treatment (LT+MAN+GSH) was associated with a very intensive accumulation of ascorbate and higher activity of enzymes involved in ascorbate regeneration (APX, MDHAR and DHAR) compared to the recalcitrant cultivar (cv. Golden Promise). Conversely to triticale, changes in GSH level and GR activity induced by various tiller pre-treatments in the studied barley cultivars suggested that glutathione did not appear to be directly involved in ME induction (Figure 5). It was confirmed by the fact that a positive effect of exogenously applied GSH on ME effectiveness has not been observed in barley, neither previously [5] nor in this study.

Ascorbate and glutathione have been localized in several cellular organelles (chloroplasts, mitochondria, peroxisomes, nuclei, cytosol and apoplast) acting independently or in concert in the Foyer–Halliwell–Asada pathway [9]. Although their essential roles are related to their antioxidant properties, and they usually respond in a compensatory manner, their functions in controlling cell division, plant growth and development seem to be interdependent and not interchangeable [6,50,51,52].

The strong reducing potential of ASC (physiologically active, anionic form of ascorbic acid) is based on the electron donating/accepting properties of the 2,3-enediol moiety [53]. It is located mainly in the cell cytoplasm, but usually about 5% of its pool is transported across the plasma membrane to the apoplast (reviewed in [9]). This fraction is believed to play a crucial role in oxidative stress signalling. The dominant role of ASC in antioxidant defence during ME induction could be explained by its substantially higher content detected in microspores of all studied plants in comparison to the level of GSH. Moreover, due to the higher affinity of APX for H_2_O_2_ in comparison to other peroxidases, the process of H_2_O_2_ removal by ASC may be more efficient than of GSH-utilising enzymes. APX activation was observed in a variety of plant species grown under stress conditions (reviewed in [9,54]). In addition, the previously observed strong seasonal effect on ME effectiveness [5] could be at least partially explained by the fact that ascorbate biosynthesis is highly sensitive to changing light conditions, especially red/far-red ratios [55,56].

The best recognized role of glutathione is also connected with its antioxidant properties determined by the cysteine sulfhydryl group (-SH) [7,57]. Acting as an electron donor/acceptor, it prevents uncontrolled, irreversible oxidation of other cellular components. However, it also plays a very important role in the protein protection through the formation of reversible, covalent disulphide bonds between the sulphur atom of GSH and protein cysteinyl residues [58]. This process of the so-called S-glutathionylation is promoted by oxidative stress, but it also occurs in cells under physiologically optimal conditions and causes specific changes in protein functions (activation or deactivation). The process is catalysed by a large family of glutathione S-transferases (GSTs), which are very diverse in structure and physiological functions. Some GSTs may also play a role as antioxidants, having peroxidase or DHAR activity ([7] and references therein). The importance of these enzymes may be underlined by the fact that certain GST transcripts have almost always been detected as upregulated in response to ME-inducing treatments [45,59,60,61,62]. Two of GST coding genes (*GSTF2, GSTA2*) were shown to be significantly upregulated in embryogenic microspores of triticale (including DH28) during the first 8 days of in vitro culture [63].

High cellular concentrations of glutathione and ascorbate have made them the major elements of redox buffering systems determining the cellular redox environment. Studies on several GSH-deficient Arabidopsis mutants [64] revealed GSH-responsive genes encoding transcription factors and other proteins involved in the regulation of cell division, redox potential and auxin signalling. Other functions of glutathione are related to redox signalling and regulation of the expression of genes determining plant growth and development. The role of glutathione in redox regulation has also been demonstrated in in vitro culture systems [65,66,67,68]. It was observed that the high redox potential (GSH/GSH+GSSG) promoted cell proliferation, whereas subsequent stages of embryo development required a more oxidized environment [65,69,70,71].

The role of the ASC/DHA ratio in cell redox state is still under discussion due to their spatial separation, since the DHA pool is mainly accumulated in the cell apoplast [6]. The results of this experiment confirmed that the level of ASC in terms of signalling and regulation seemed to be more important than the ASC/DHA ratio. This was probably due to other important roles of ASC, which is a cofactor of several enzymes involved in photoprotection, biosynthesis of plant growth regulators, pigments and secondary metabolites [8].

## 5. Conclusions

These observations support our hypothesis that ROS generation and antioxidant defence are critical for successful ME induction. The analysis of our data suggests that the very subtle, dynamic homeostasis between ROS production and scavenging determines the effectiveness of microspore reprogramming towards sporophytic development. Stress treatments effective for ME induction induce ROS generation and simultaneously enhance antioxidant defence in a balanced manner. Genetic and physiological determination of the antioxidant defence system could explain the observed variation in plant responsiveness to various stresses applied as triggers for ME induction. It appears to be based on stress tolerance mechanisms that have developed during the evolution of individual species in response to the most common environmental challenges. In addition to the function of basic antioxidant enzymes like SOD and CAT, enzymes involved in the Foyer–Halliwell–Asada cycle also seem to play an important role in the successful microspore reprogramming. The roles played by the main LMW antioxidants—ascorbate and glutathione—are much more complex and genotype-specific. They are based not only on their antioxidant capacity, but also on their potential key role in cell redox signalling and regulation of cell growth and differentiation. The highly complex multifactorial network of mutual interactions makes the development of effective ME induction procedures in certain economically important crop species still a great challenge for researchers and breeders. To overcome this challenge, more research is needed into the mechanism of microspore reprogramming. A very interesting and almost non-recognized problem is the source and subcellular localization of ROS generation and its interaction with other signalling molecules like Ca^2+^, plant growth regulators or reactive nitrogen species [47]. Other various possibilities are the involvement of polyamine oxidases (PAOs) and/or NADPH-oxidase as enzymes contributing to H_2_O_2_ accumulation in response to abiotic stress [72] and generative development of microspores [73]. The direction of further research will be based however, on the results of RNAseq analysis which will show the most probable and promising objects of interest.

## Figures and Tables

**Table 1:** The effect of tillers pre-treatment on microspore yield (×10^3^), viability (%) and the effectiveness of microspore embryogenesis (ME) induction (ELS/spike) in isolated microspore cultures of two DH lines of triticale and two cultivars of barley. Data represent means from 3–5 biological replications (isolations) ± SE. Statistical analysis was performed separately for triticale and barley. Data marked with different letters differ significantly according to the Duncan test (*p* ≤ 0.05); ns, not significant.

Plant Material	Treatment	Microspore Yield	Viability	ME Induction
DH19	LT (control)	111.0 ± 11 ^ns^	14.3 ±1 ^cd^	0
	LT+MAN	103.5 ± 11	6.9 ± 1 ^d^	0
	LT+GSH	88.5 ± 6	17.8 ± 2 ^bc^	0
	LT+MAN+GSH	116.5 ± 6	5.9 ± 1 ^d^	0.7 ± 0.7
	mean	104.8 ± 5	11.7 ± 1	0.2 ± 0.2
DH28	LT (control)	99.2 ± 6 ^ns^	30.3 ± 3 ^a^	17 ± 6 ^ns^
	LT+MAN	87.1 ± 10	17.4 ± 5 ^bc^	32 ± 11
	LT+GSH	86.3 ± 67	29.4 ± 2 ^a^	12 ± 5
	LT+MAN+GSH	81.2 ± 5	24.0 ± 3 ^ab^	31 ± 18
	mean	88.4 ± 3	26.1 ±2	22± 5
cv. Golden Promise	LT (control)	29 ± 4 ^ns^	51 ± 3 ^b^	2 ± 0.9 ^c^
	LT+MAN	27 ± 5	57 ± 3 ^ab^	15 ± 7 ^c^
	LT+GSH	26 ± 5	53 ± 3 ^b^	2 ± 1 ^c^
	LT+MAN+GSH	25 ± 4	58 ± 3 ^ab^	12 ± 8 ^c^
	mean	26.1 ± 2	54.7 ± 1	8 ± 2
cv. Igri	LT (control)	21 ± 6 ^ns^	65 ± 2 ^a^	414 ± 121 ^bc^
	LT+MAN	25 ± 7	64 ± 2 ^a^	1069 ± 407 ^b^
	LT+GSH	28 ± 8	57 ± 3 ^ab^	543 ± 296 ^bc^
	LT+MAN+GSH	32 ± 9	59 ± 2 ^ab^	2047 ± 622 ^a^
	mean	30.4 ± 5	61.6 ± 1	1073 ± 277

ELS—embryo-like structures; LT—low-temperature tiller pre-treatment (21 days at 4 °C); LT+GSH—low-temperature tiller pre-treatment combined with the application of 0.3 mmol·dm^−3^ reduced glutathione during the last 4 days before microspore isolation; LT+MAN—low-temperature tiller pre-treatment combined with the application of 0.3 mol·dm^−3^ mannitol during the last 4 days before microspore isolation; LT+MAN+GSH—low-temperature tiller pre-treatment combined with the application of reduced 0.3 mmol·dm^−3^ glutathione and 0.3 mol·dm^−3^ mannitol during the last 4 days before microspore isolation.

**Table 2:** The sources of variance for content of reduced ascorbate (ASC), reduced glutathione (GSH), total activity of low molecular weight (LMW) antioxidants, activity od ascorbate peroxidase (APX), monodehydroascorbate reductase (MDHAR), dehydroascorbate reductase (DHAR) and glutathione reductase (GR) were as follows: four plant genotypes, four tillers pre-treatments, and interaction between plant genotype and the treatment.

Parameter	Variable	MS	*F*	*p*
ASC	(1) Plant genotype	118 ^E4^	179.5	***
	(2) Tillers pre-treatment	147 ^E3^	22.4	***
	(1) × (2)	164 ^E3^	24.9	***
GSH	(1) Plant genotype	199 ^E2^	172.0	***
	(2) Tillers pre-treatment	252	2.2	ns
	(1) × (2)	826	7.1	***
LMW antioxidants	(1) Plant genotype	3.564	22.13	***
	(2) Tillers pre-treatment	5.635	34.99	***
	(1) × (2)	2.002	12.43	***
APX	(1) Plant genotype	887 ^E3^	216.6	***
	(2) Tillers pre-treatment	294 ^E3^	70.2	***
	(1) × (2)	279 ^E3^	66.4	***
MDHAR	(1) Plant genotype	107 ^E2^	61.9	**
	(2) Tillers pre-treatment	216 ^E2^	125.2	**
	(1) × (2)	9349	54.3	**
DHAR	(1) Plant genotype	468 ^E2^	576.4	***
	(2) Tillers pre-treatment	2113	26.0	***
	(1) × (2)	2181	26.8	***
GR	(1) Plant genotype	7468	91.21	***
	(2) Tillers pre-treatment	1298	15.85	***
	(1) × (2)	398	4.86	***

MS—mean squares, an estimate of variance of a variable or interaction between variables; *F*—*F* calculated as variance of a variable or interaction between variables divided by error variance of the experiment; *p*—error probability for rejection of the null hypothesis about the significance of variability within a variable or interaction between variables: ***, **, ns—significant at *p* ≤ 0.001, *p* ≤ 0.01, not significant, respectively; E—scientific notation (times ten raised to the power of two, three, etc.)

## Data Availability

Data is contained within the article and supplementary material.

## Supplementary Materials

The following are available online at https://www.mdpi.com/article/10.3390/antiox10081254/s1, Figure S1: In situ histochemical detection of superoxide anion (A–C) and hydrogen peroxide (D–F) in randomly selected samples of isolated microspores at the early stages of microspore embryogenesis.

## Abbreviations

ASC—reduced ascorbate; APX—ascorbate peroxidase; CAT—catalase; DHA—dehydroascorbate; DHAR—dehydroascorbate reductase; ELS—embryo-like structure; GR—glutathione reductase; GSH—reduced glutathione; GSSG—glutathione disulphide; HS—Hoagland’s nutrient solution; LMW—low molecular weight antioxidants; LT—low temperature tillers treatment; LT+GSH—tillers treatment with low temperature and 0.3 mM GSH; LT+MAN—tillers treatment with low temperature and 0.3 M mannitol; LT+MAN+GSH— tillers treatment with low temperature, 0.3 M mannitol and 0.3 mM GSH; MDHA—monodehydroascorbate; MDHAR—monodehydroascorbate reductase; ME—microspore embryogenesis; NADH—reduced nicotinamide adenine dinucleotide; NADPH—nicotinamide adenine dinucleotide phosphate; ROS—reactive oxygen species; SOD—superoxide dismutase.
