# Supplementary material for: Impact of Ascorbate—Glutathione Cycle Components on the Effectiveness of Embryogenesis Induction in Isolated Microspore Cultures of Barley and Triticale
Source: Antioxidants (Basel). 2021 Aug 5;10(8):1254. doi: 10.3390/antiox10081254 (PMC8389252; doi:10.3390/antiox10081254)
Supplement: Supplementary file 1 [file antioxidants-10-01254-s001.zip › antioxidants-1273749-supplementary.pdf]

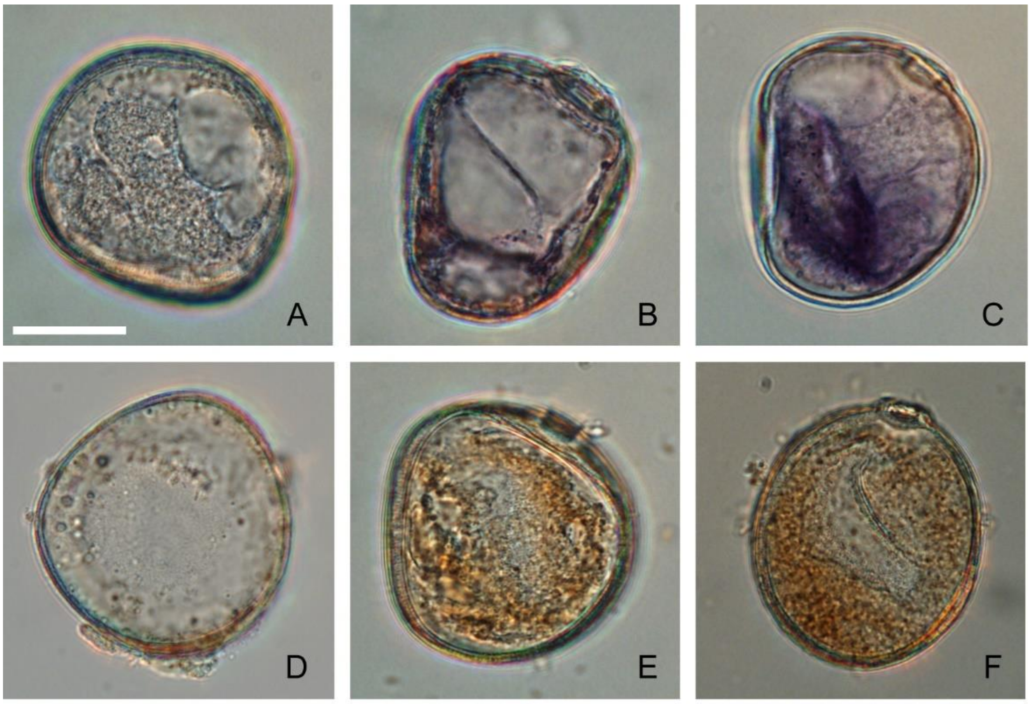

Figure S1. *In situ* histochemical detection of superoxide anion (**A**,**B**,**C**) and hydrogen peroxide (**D**,**E**,**F**) in randomly selected samples of isolated microspores at the early stages of microspore embryogenesis. Variation in patterns of superoxide anion and hydrogen peroxide distribution in the cytoplasm revealed by NBT (**B**,**C**) and DAB staining (**E**,**F**) in triticale microspores (DH28) induced to sporophytic development by low temperature tillers pre-treatment (LT; control). Negative controls for NBT (**A**) and DAB (**D**) staining. Blue staining demonstrates the spatial distribution of superoxide anions. Reddish-brown staining demonstrates the spatial distribution of hydrogen peroxide. Bar: 20 μm.
